# Cryo-EM: the revolution continues

**DOI:** 10.1107/S2052252526003842

**Published:** 2026-06-08

**Authors:** Sriram Subramaniam, Werner Kühlbrandt, Richard Henderson

**Affiliations:** a Department of Biochemistry and Molecular Biology University of British Columbia Vancouver BC V6T 1Z3 Canada; b Department of Structural Biology Max Planck Institute of Biophysics Frankfurt60438 Germany; c Department of Biochemistry Goethe University Frankfurt am Main Germany; d MRC Laboratory of Molecular Biology, CambridgeCB2 0QH, United Kingdom; University of Liverpool, United Kingdom

**Keywords:** 3D reconstruction and image processing, automation, electron tomography, single-particle cryo-EM, advances in microscope hardware, structural biology

## Abstract

The near-universal adoption of electron cryo-microscopy (cryo-EM) by structural and cell biologists has led to exponential growth of the field, especially over the last two decades, with a doubling in the number of deposited electron-microscopy density maps every 2.5 years. This exponential growth has changed in recent years to become linear. Cryo-EM methods are now able to contribute to our structural understanding of biological complexity ranging from atomic resolution maps of assemblies of multiprotein complexes to *in situ* investigation of protein structures in the context of intact cells, snapshots of multiple conformations and insights into fundamental chemical mechanisms underlying biological function. Combined with dramatic advances in artificial intelligence, the increasing democratization and implementation of cryo-EM appears poised to drive a new revolution in digital biology.

## Abstract 2

The increasing democratization and implementation of electron cryo-microscopy appears poised to drive a new revolution in digital structural biology.

## 1. Introduction

The determination of the three-dimensional structure of bacteriorhodopsin 50 years ago by imaging proteins with electron microscopy was the first demonstration that this approach could be valuable as a tool in structural biology. The 7 Å resolution (Henderson & Unwin, 1975 ▸) obtained by combining images of two-dimensional crystals of bacterio­rhodopsin was extended further by imaging at cryogenic temperatures, resulting in a report of the structure at 3.5 Å resolution (Henderson *et al.*, 1990 ▸), the first use of cryogenic electron microscopy (cryo-EM) for the determination of protein structures.

**Figure 1:**
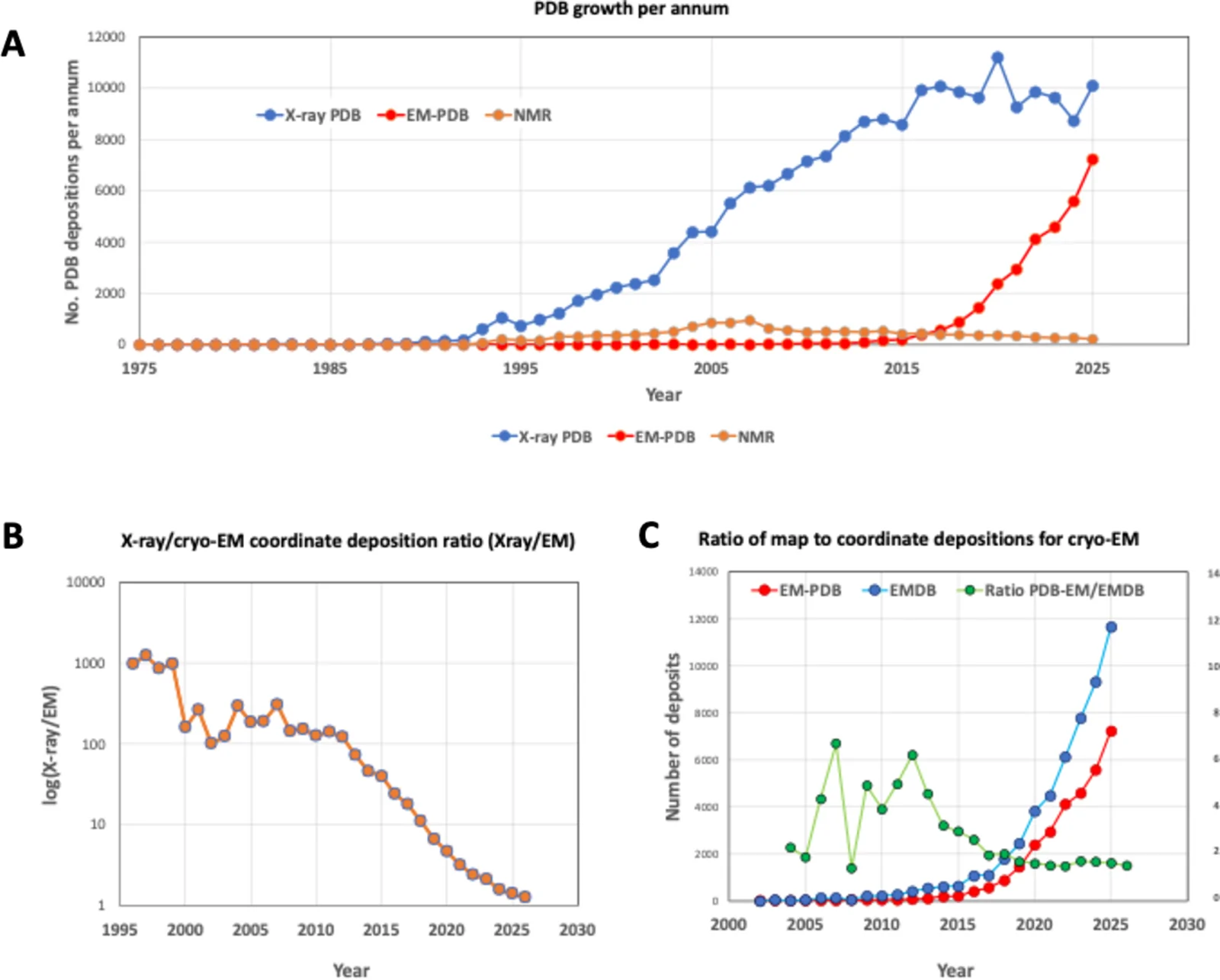
Plot of the number of depositions in the EMDB and the PDB over the last 50 years. (*A*) Annual growth in PDB structures from X-ray crystallography (blue), cryo-EM (red) and NMR (brown). (*B*) Change in ratio of depositions from X-ray crystallography and cryo-EM over time. (*C*) Growth in the number of depositions of cryo-EM maps (blue) and cryo-EM-derived PDB coordinates (red), and the change in the ratio (green) over time. For (*C*), the right axis represents the ratio. The data are derived from information available at the EMDB (https://www.ebi.ac.uk/emdb).

Fig. 1 ▸ shows the growth of cryo-EM maps and PDB models deposited in the Electron Microscopy Data Bank (EMDB) and the Protein Data Bank (PDB). The depositions based on X-ray crystallography grew exponentially between 1985 and 1995 then linearly from 1995 until 2015 after which there has been a plateau at ∼10 000 PDB X-ray depositions annually except for a slight spike due to depositions in 2020 related to the SARS-CoV-2 virus. The use of X-ray crystallography for structure determination depends on the generation of three-dimensional crystals that diffract to high enough resolutions, and this is generally easier for smaller proteins such as enzymes or individual domains of longer polypeptides. In contrast, single-particle cryo-EM structures require protein/nucleic acid complexes that are large enough to allow determination of particle orientations in projection cryo-EM images. Structure determination by cryo-EM remains challenging for smaller proteins (<50 kDa), which account for less than 2% of all reconstructions deposited in the EMDB. However, depositions of structures for 50–100 kDa proteins (currently ∼6.5%) and 100–250 kDa proteins (∼32%) are increasing steadily (https://www.ebi.ac.uk/emdb).

There is not yet any sign of a plateau in cryo-EM productivity with ∼7200 cryo-EM PDB depositions in 2025, close to the ∼10 000 deposited X-ray structures over the same period [Figs. 1 ▸(*A*) and 1 ▸(*B*)]. There were also ∼12 000 3D map depositions from cryo-EM. This ratio of ∼1.5× between EMDB map depositions and PDB coordinate depositions [Fig. 1 ▸(*C*)] has remained constant for the last ten years since the beginning of the ‘resolution revolution’ (Kühlbrandt, 2014 ▸). At some point, the number of annual depositions from cryo-EM will likely hit a plateau, the way depositions from X-ray crystallography have plateaued, but that seems some distance away. In contrast to X-ray structure depositions, there are often several EMDB and PDB depositions from the same cryo-EM dataset obtained via 3D classification of different structural states. Another interesting aspect of cryo-EM depositions is that there are instances where structures have been determined from heterogeneous mixtures where the complex of interest is present alongside other complexes (Spaulding *et al.*, 2018 ▸) or where multiple structures are determined from partially purified or unpurified samples (Wang *et al.*, 2024 ▸). There have also been cryo-EM structures deposited for the same proteins such as respiratory complexes where there are ∼300 entries of varying qualities from different organisms over the years with resolutions ranging from ∼30 Å to ∼1.8 Å, with increasing resolution driven by improvements in biochemistry, choice of organism, microscopy and image processing.

## 2. Continuing improvements in quality and complexity of cryo-EM structures

Until 2013, most cryo-EM structure depositions came from studies of large and mostly symmetric complexes with sizes >500 kDa, but there has been rapid growth in subsequent years in deposition of structures of proteins and protein complexes spanning a wide range of sizes.

The structures being deposited now come from increasingly complex assemblies such as GPCR–G protein complexes, amyloid fibres and dynamic multi-protein complexes, although in many cases there are high-resolution X-ray structures for individual domains of these complexes. The critical value of using cryo-EM for accelerating drug design (Subramaniam *et al.*, 2016 ▸) and validating the binding mode of small and large therapeutic molecules is now well established with successful application to challenging problems such as mapping the binding of molecular glue degraders to complexes of cellular targets with E3 ligases (Dippon *et al.*, 2026 ▸; Watson *et al.*, 2022 ▸). Native membrane protein complexes such as the human erythrocyte ankyrin-1 complex are essentially intractable by X-ray crystallography methods but have been analysed at high resolution using cryo-EM (Vallese *et al.*, 2022 ▸; Xia *et al.*, 2022 ▸). The ability to obtain structures of native integral membrane protein complexes is especially exciting, with more than ten times as many structures deposited using cryo-EM methods as compared with X-ray crystallography. In some instances, cryo-EM has also been used for structure determination of smaller proteins such as the 32 kDa haemoglobin dimer extracted from a haemoglobin tetramer dataset (Kim *et al.*, 2025 ▸) and the 40 kDa Aca2–RNA complex (Kimanius *et al.*, 2024 ▸).

**Figure 2:**
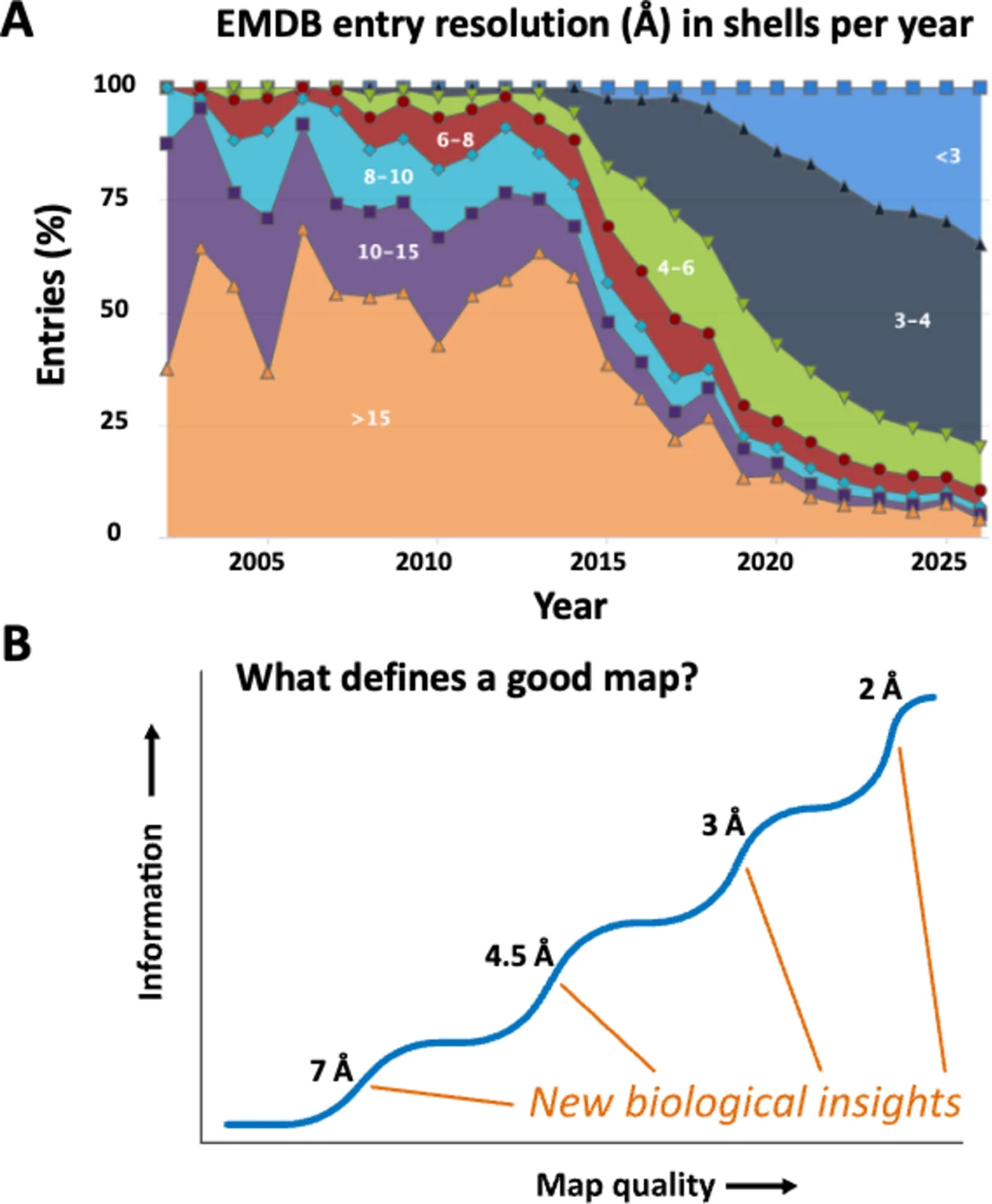
(*A*) Plot of the change over time in the distribution of resolutions in deposited cryo-EM structures. (*B*) Schematic representation of the concept that there is a progressive increase in the amount of information that can be derived from cryo-EM maps as the resolution improves, with inflection points at selected resolutions where new structural features can be discerned: α-helices at 7 Å, β-strands at 4.5 Å, side chains at 3 Å, and water molecules and other high-resolution features at 2 Å and beyond.

Over the years, there has been a progressive increase in the resolutions of the structures reported, with more than a quarter of all structures being reported currently at resolutions better than 3 Å [Fig. 2 ▸(*A*)]. There has been a steady increase in the number of structures reported at resolutions better than ∼2 Å starting with glutamate dehydrogenase at 1.8 Å (Merk *et al.*, 2016 ▸) a decade ago, extending now to several challenging G-protein complexes such as the cholecystokinin-1-receptor–G-protein-nanobody complex resolved to 1.95 Å (Mobbs *et al.*, 2021 ▸). The highest resolutions recently reported are for spinach rubisco in complex with transition-state analogue 2-carboxyarabinitol-1,5-bisphosphate at 1.25 Å (Croy *et al.*, 2025 ▸), and numerous structures for the well ordered apoferritin protein complex, *e.g.* 1.24 Å by Danev *et al.* (2025 ▸) following earlier high-resolution structures of apoferritin reported a few years ago (Nakane *et al.*, 2020 ▸; Yip *et al.*, 2020 ▸), including a report of resolution estimated to be as high as 1.09 Å (Küçükoğlu *et al.*, 2024 ▸).

Resolution values are, however, merely single numbers that reflect the quality at which the best-ordered regions of the protein can be visualized. Inspection of the map quality in different regions provides a more accurate measure of the information content in any particular region of the protein, with meaningful inflection points at ∼7 Å when α-helices are resolved, ∼4.5 Å when β-strands are resolved, ∼3 Å when most side chains are resolved, and ∼2 Å when water molecules and other high-resolution features can be gleaned [illustrated schematically in Fig. 2 ▸(*B*)]. In a few rare cases, high-resolution information has provided important insights beyond structure and extending into chemical mechanisms, for instance in the structural analysis of fatty acid synthase (Singh *et al.*, 2023 ▸) where the higher (1.9 Å) resolution reveals a 22° kink in the isoalloxazine ring system of flavin mononucleotide indicating that it must be in the reduced state. Another recent example is the convincing demonstration of the chemical structure of the iminosugar glycosyrin captured in complex with lacz β-galactosidase at ∼1.4 Å resolution (Sanguankiattichai *et al.*, 2025 ▸).

There are now excellent tools such as *ResMap* (Kucukelbir *et al.*, 2014 ▸) and *CryoRes* (Dai *et al.*, 2023 ▸) that allow determination of the variation in resolution across different regions of the protein or protein complex. These measures provide valuable additional information especially on conformational heterogeneity, which is an important determinant of the resolution that can be achieved, as is the overall stability of the protein. In some instances, selected regions of the structure are at lower resolution because they are smaller domains attached by a flexible hinge to larger domains that drive particle alignment for 3D reconstruction, but this can be offset by focusing the 3D alignment on the smaller domain (Mannar *et al.*, 2022 ▸). In some other instances, the use of powerful tools for 3D classification of images allows separation into multiple conformations, each at higher quality than the composite map that represents the average of all of the conformations, for example in structural studies of the flagellar motor (Singh & Iverson, 2025 ▸). These methods need to be used with caution though, given the potential for interpreting noise in the images as being indicative of evidence for observing true conformations.

In addition to visualizing large and dynamic protein complexes, there is growing interest in extending the methods to the study of biological assemblies containing only RNA, which have eluded analysis so far at high resolution. The determination of cryo-EM structures of RNA-only complexes such as ROOL and GOLLD at resolutions of ∼2–3 Å reveal higher-order architectures stabilized by an array of tertiary motifs underlying the conserved principles of RNA self-assembly (Kretsch *et al.*, 2025 ▸; Zhang *et al.*, 2025 ▸), providing hope that other similar structures may shed new light into the architecture of non-coding RNA molecules.

## 3. Progress in cryo-tomography (cryo-ET)

The vast majority of high-resolution maps and structures determined by cryo-EM so far are of proteins and protein complexes either produced by recombinant DNA technology or isolated and purified from the living cells and tissues where they perform their functions. These structures of isolated particles are often (perhaps misleadingly) referred to as ‘single-particle’ structures, although they are averages of hundreds of thousands, even millions, of identical or near-identical molecules. However, cryo-EM is not limited to isolated particles in aqueous suspension. As one of the method’s greatest strengths, it can examine and determine the structures of these particles directly by electron cryo-tomography (cryo-ET) in their cellular context *in situ*, provided that they are large enough to be recognized. Most often the molecular assemblies are identified in tomographic volumes generated by reconstructing tomographic tilt series of thin vitreous lamellae excised with an ion or plasma beam from their cells or tissues.

Cryo-ET has allowed visualization of the interior of cells and tissues at unprecedented resolution (Nogales & Mahamid, 2024 ▸). In addition to the visualization of overall subcellular architecture, in a process called sub-tomogram averaging, small subvolumes containing a particular molecular assembly are cut out computationally. The individual subvolumes are then aligned and averaged, much as in single-particle cryo-EM, using essentially the same approach to determine three-dimensional structures (Liu *et al.*, 2008 ▸). This approach works well with viral glycoproteins and cellular compartments that contain large numbers of well characterized macromolecular assemblies such as mitochondria. In this way, the *in situ* structure of a mitochondrial ATP synthase has been determined at up to 4.2 Å, resolving individual large side chains within α-helices. By 3D classification, six distinct rotary states were resolved, each with several rotary substates (Dietrich *et al.*, 2024 ▸). The resolution is limited by the size and number of particles that can be identified and averaged. With ribosomes in tomographic volumes of human tissue culture cells, resolutions approaching ∼2 Å are being achieved (Xing *et al.*, 2023 ▸; Zheng *et al.*, 2025 ▸), while prokaryotic S-layers from whole archeal cells have been visualized at ∼3.3 Å (von Kügelgen *et al.*, 2024 ▸).

As an alternative to reconstructing and averaging 3D volumes from tomographic tilt series, it is sometimes possible to image organelles such as mitochondria directly on a cryo-EM grid and then to select and classify large particles with characteristic views by single-particle methods. This procedure has yielded structures of several different respiratory chain supercomplexes within their native mitochondrial membranes (Zheng *et al.*, 2024 ▸).

## 4. Hardware and software advances

There have been significant advances in detector technology with bigger, faster detectors with improved detective quantum efficiency (DQE). At 300 keV, the Gatan K2 and K3 detectors have been a mainstay for many years and have been used as a base for development of the Alpine and Alpine Vista, which have improved performance at lower electron energy (Chan *et al.*, 2024 ▸). The Thermo Fisher Falcon 4i that was introduced in 2022 has also progressively grown in popularity. The Thermo Fisher Selectris energy filter has also been available since 2023 and has provided a valuable alternative to the Gatan Quantum series. The Gatan K3 detector is larger and faster than the K2, and even with correlated double sampling to reduce readout noise, it still has the fastest frame rate. The Falcon 4i detector has half the frame rate of the K3 but has better signal-to-noise discrimination for the detected single electron events. The new Direct Electron Apollo detector is another contender, offering high performance with a novel sensor that allows electron counting in hardware. Future improvements could come from bigger and faster detectors without loss of performance in terms of DQE.

In terms of electron optics, field emission guns (FEGs), which provide greatly improved spatial and temporal coherence due to reduced source size and energy spread compared with the older thermionic electron sources, have been improved by the introduction of cold FEGs (Ricolleau *et al.*, 2013 ▸). Finally, through the introduction of aberration-free image-shift alignment and procedures (https://documents.thermofisher.com/TFS-Assets/MSD/Application-Notes/fringe-free-imaging-an016.pdf), the ability to collect more images or movies per hour has allowed structures to be determined more quickly or at higher resolution than ever.

The potential for improved images by cooling the specimen to a lower temperature, near to that of liquid helium, has also been demonstrated both through electron diffraction of 2D crystals and from single-particle cryo-EM images in ice (Dickerson *et al.*, 2025 ▸; Naydenova *et al.*, 2022 ▸). The rate of development of structural disorder in the samples was reduced by ∼1.5-fold compared with more typical specimen temperatures of 80 K. Hardware advances have also targeted the development of 100 keV detectors optimized for lower-energy electrons, which carry more useful information per unit of radiation damage than 300 keV electrons, resulting in potentially more useful high-resolution cryo-EM structures (McMullan *et al.*, 2023 ▸).

Some of the developments on the horizon include phase plates that offer alternative approaches to generation of improved low-resolution contrast based on ponderomotive retardation of electrons by a focused optical laser beam (Axelrod *et al.*, 2024 ▸). The improved low-resolution contrast will help more accurate determination of orientations in projection images for 3D reconstruction in cryo-EM and cryo-ET. The use of aberration-corrected imaging, especially combined *C*_c_ and *C*_s_ correction, has been available in materials science electron microscopy for many years, but its implementation in cryo-EM is still very recent (Wu *et al.*, 2025 ▸). Combining these advances with a laser phase plate or the use of methods such as electron ptychography (Küçükoğlu *et al.*, 2024 ▸) – a coherent diffractive imaging technique using 4D scanning transmission electron microscopy – offers the promise of higher contrast that may be useful for applications in both cryo-EM and cryo-ET. Impressive increases in the speed of structure determination by cryo-EM have also resulted from advances in software for image processing over the last decade combined with the advent of more powerful computing platforms. While *RELION* (Scheres, 2012 ▸) and *cryoSPARC* (Punjani *et al.*, 2017 ▸) have been the main programs used to determine the structures that have been deposited in the PDB, various modules developed for sub-tasks such as CTF determination and for model refinement such as *Coot* (Emsley *et al.*, 2010 ▸) provide a diverse set of tools to enable efficient end-to-end workflows for data processing.

Advances in software for image analysis are also providing powerful new tools to understand protein dynamics. The analysis of conformational heterogeneity is an area of considerable current interest in the ongoing development of numerous new software packages under development. These include a variety of methods that leverage representation of the data in a latent space to derive more accurate representations of conformation, such as *HetSIREN*, a deep-learning-based method (Herreros *et al.*, 2025 ▸). *RECOVAR* uses principal component analysis computed via regularized covariance estimation (Gilles & Singer, 2025 ▸), *cryoDRGN* (Zhong *et al.*, 2021 ▸) leverages deep neural networks and *ManifoldEM* (Ojha *et al.*, 2025 ▸) uses a geometric machine-learning-based approach to address continuous conformational heterogeneity. Many other similar tools are also being developed. An important caveat to these methods is that while each may reveal the presence of conformational heterogeneity, there remain significant challenges in translating these discoveries into tangible mechanistic information, even in relatively simple cases such as the TrpV1 ion channel (Astore *et al.*, 2025 ▸).

A key challenge in cryo-ET is to identify the macromolecular assemblies in the low-contrast tomographic volumes. Correlative light and electron microscopy with fluorescently labelled targets is being used to overcome this problem, and computational template matching and models generated by *AlphaFold* are increasingly successful (Kovalevskiy *et al.*, 2024 ▸; Mosalaganti *et al.*, 2022 ▸). Another issue is the small fraction of the cellular volume that can be imaged in 3D by focused-ion-beam (FIB) milling and cryo-ET. Serial FIB lamellae or even serial vitrified sections cut with a diamond knife, a technique known as CEMOVIS (Elferich *et al.*, 2025 ▸), are being explored for increasing the accessible cellular volume and providing context.

## 5. Cryo-EM, cryo-ET and AI

The arrival of *AlphaFold* in 2021 heralded a paradigm shift in structural biology (Jumper *et al.*, 2021 ▸). The possibility to produce atomic models using *AlphaFold* (more recently *AlphaFold 3*) for any unknown protein or protein complex even before an experiment is carried out results in a dramatic lowering of the entry barriers into structural biology for most scientists who are not direct practitioners of X-ray crystallography, NMR spectroscopy or cryo-EM. The availability of the predictions means that functional studies involving proteins can now start with a structural model. Testing of a series of predictions that can validate, repudiate or refine the structural hypothesis then becomes the principal goal of the experiments that are carried out. The science of structural biology, which is aimed at understanding biological function through structure, could thus advance by means of a highly effective iteration between structure prediction and experimental validation or correction (Subramaniam & Kleywegt, 2022 ▸).

There are valid concerns about the accuracy of the structures predicted by *AlphaFold* based on comparison with high-resolution crystallographic structures, as noted recently (Terwilliger *et al.*, 2024 ▸). However, there are also limitations to viewing accuracy solely through the lens of the quality of fit of the model to the experimental electron density of a crystallized protein (Subramaniam, 2024 ▸). It is conceivable that neither the *AlphaFold* prediction nor the crystal structure may reflect the true conformation of the protein under near-physiological conditions. Fortunately, cryo-EM and cryo-ET methods provide a unique opportunity to explore this landscape, and the integration with AI-driven predictions to build *in silico* models for large subcellular assemblies promises to be one of the most fertile areas at the interface between structural and cell biology in the coming decade, further fueling the increasing trend towards digitization of biology.

Is it possible that the rapid advances in AI will make AI-generated structure prediction the new normal and eliminate the need for experimental structure determination? This does not seem likely, at least in the near future. The ability to generate many plausible models in fact further increases the importance of validation of the models by incisive experimental approaches. However, there is need for much greater throughput in structure determination by cryo-EM and cryo-ET to keep pace with the need for very large data sets to train foundational models that can provide more accurate structural predictions. It remains to be seen whether significant increases in speed of obtaining cryo-EM and cryo-ET structures can be achieved in the coming decade. One thing seems clear though for now: AI is not yet at a stage where it can replace experimental structural biology, but our ability to integrate AI with high-throughput experimental data will almost certainly guide the future of structural biology.
